# FM19G11 inhibits O^6^‐methylguanine DNA‐methyltransferase expression under both hypoxic and normoxic conditions

**DOI:** 10.1002/cam4.1551

**Published:** 2018-05-15

**Authors:** Chao‐guo You, Han‐song Sheng, Chao‐ran Xie, Nu Zhang, Xue‐sheng Zheng

**Affiliations:** ^1^ Department of Neurosurgery Xinhua Hospital Affiliated to Shanghai JiaoTong University School of Medicine Shanghai China; ^2^ Department of Neurosurgery The Second Affiliated Hospital of Wenzhou Medical University Wenzhou China

**Keywords:** FM19G11, glioblastoma, O^6^‐methylguanine DNA‐methyltransferase

## Abstract

FM19G11 is a small molecular agent that inhibits hypoxia‐inducible factor‐1‐alpha (HIF‐1α) and other signaling pathways. In this study, we characterized the modulating effects of FM19G11 on O^6^‐methylguanine DNA‐methyltransferase (MGMT), the main regulator of temozolomide (TMZ) resistance in glioblastomas. This study included 2 MGMT‐positive cell lines (GBM‐XD and T98G). MGMT promoter methylation status, mRNA abundance, and protein levels were determined before and after FM19G11 treatment, and the roles of various signaling pathways were characterized. Under hypoxic conditions, MGMT mRNA and protein levels were significantly downregulated by FM19G11 via the HIF‐1α pathway in both GBM‐XD and T98G cells. In normoxic culture, T98G cells were strongly positive for MGMT, and MGMT expression was substantially downregulated by FM19G11 via the NF‐κB pathway. In addition, TMZ resistance was reversed by treatment with FM19G11. Meanwhile, FM19G11 has no cytotoxicity at its effective dose. FM19G11 could potentially be used to counteract TMZ resistance in MGMT‐positive glioblastomas.

## 1 INTRODUCTION

Glioblastoma (GBM) is the most common primary malignant brain tumor in adults, accounting for approximately 39% of central nervous system neoplasms. Treatment typically includes maximal safe resection plus postoperative adjuvant ionizing radiation and chemotherapy with temozolomide (TMZ). In 2005, Stupp et al^1^ reported a 2.5‐month overall survival (OS) benefit with the addition of the alkylating agent TMZ to surgery and radiation; thus, this treatment plan is termed Stupp's regimen.

Although the median survival time of patients with GBM receiving the Stupp regimen is only 14.6 months, it is noteworthy that patients with low O^6^‐methylguanine DNA‐methyltransferase (MGMT) expression benefit more from TMZ chemotherapy than patients with high MGMT expression.^2^ According to a recent study, the median OS of a low MGMT group of patients with GBM receiving the Stupp regimen was 21.8 months, while that of a high MGMT group was only 13.1 months.^3^

TMZ exhibits cytotoxicity mostly by methylating the O^6^ position of guanine and then causing tumor cell apoptosis. MGMT directly removes the methyl group at the O^6^ position of guanine and thus reverses the cytotoxic effects of TMZ.^3^ Therefore, inhibition of MGMT expression may help to overcome TMZ resistance in GBM.^2^, ^4^

If the MGMT promoter is methylated, the gene is silenced.^2^ Otherwise, the gene will be expressed, and the expression efficiency is modulated by many mechanisms, including the hypoxia‐inducible factor‐1‐alpha^5^ (HIF‐1α), NF‐κB,^6^ and WNT/β‐catenin pathways.^7^

Of late, Tang et al^5^ reported that the HIF‐1α inhibitor 2‐methoxyestradiol (2‐ME) downregulated MGMT expression under hypoxic conditions. However, 2‐ME is highly cytotoxic, which limits further investigation in preclinical settings, and it only exerts an effect under hypoxic conditions.^5^ FM19G11 is a novel small molecule (molecular weight: 463.40 g/mol) HIF‐1α inhibitor with an effective dose in the nanomolar range, and it is very safe at concentrations lower than 30 μmol/L.^8^ In addition, FM19G11 modulates other signaling pathways, including mTOR^9^ and PI3K/Akt/eNOS,^10^ when the HIF‐1α pathway is inactivated under normoxic conditions. Therefore, we hypothesized that FM19G11 suppresses MGMT expression under both hypoxic and normoxic conditions through different mechanisms. This study was performed to test this hypothesis.

## 2 METHODS

### 2.1 GBM cell culture

The T98G GBM cell line was provided and authenticated by the Shanghai Institute of Biochemistry and Cell Biology, Shanghai, China. GBM‐XD is a primary cell strain derived from the surgical specimen of a patient with World Health Organization grade IV GBM undergoing resection in accordance with a protocol approved by the Ethics Committee of our hospital and with prior informed consent from the patient. The culture medium was composed of DMEM (Life Technologies/GIBCO, Carlsbad, CA, USA) and 10% fetal bovine serum (FBS; Life Technologies/GIBCO). The cells were cultured at a density of 1 × 10^5^ cells/mL. For normoxic culture, the cells were incubated at 37°C with 95% air, 5% CO_2_, and 100% humidity. For hypoxic culture, the cells were incubated at 37°C with 1% O_2_, 5% CO_2_, and 100% humidity.

### 2.2 Cell viability assay

The cytotoxic effects of TMZ (Sigma‐Aldrich, St. Louis, MO, USA) and FM19G11 (Sigma‐Aldrich) were measured using the CellTiter 96 AQueous Non‐Radioactive Cell Proliferation Assay (Promega Corp., Madison, WI, USA) following the manufacturer's protocol. In brief, GBM‐XD and T98G cells were seeded in 96‐well flat‐bottom plates at 5000 cells/well, cultured in DMEM supplemented with 10% FBS, and then treated with TMZ and/or FM19G11, or DMSO as a control. A mixture of 100 μL phenazine methosulfate (PMS) and 2 mL of [3‐(4,5‐dimethylthiazol‐2‐yl)‐5‐(3‐carboxymethoxyphenyl)‐2‐(4‐sulfophenyl)‐2H‐tetrazolium (MTS) reagent was freshly prepared. Next, 20 μL of MTS/PMS mix reagent was added to 100 μl of media per well, and the cells were incubated at 37°C for 2 hours. The optical density (OD) was measured at 490 nm with a spectrophotometer. Relative cell viability was expressed as the ratio of the OD of TMZ and/or FM19G11‐treated cells to the OD of control cells.

### 2.3 Western blotting

Cells were rinsed with phosphate‐buffered saline (PBS), lyzed in radioimmunoprecipitation assay (RIPA) buffer (150 mmol/L NaCl, 25 mmol/L Tris [pH 7.4], 1% Triton X‐100, 0.5% sodium dodecyl sulfate [SDS], and 5 mmol/L EDTA), and cleared by centrifugation in a microfuge at 20 000 × *g* for 15 minutes. The protein concentration was determined using bovine serum albumin as the standard. Equal amounts of protein were used for SDS polyacrylamide gel electrophoresis (SDS‐PAGE). Gels were electroblotted onto nitrocellulose membranes that were blocked for 1 hours with 5% nonfat dry milk in Tris‐buffered saline (TBS) containing 0.1% Tween‐20. The membranes were then incubated at 4°C overnight with primary antibodies. After rinsing in PBS, the membranes were incubated at room temperature for 1 hours with peroxidase‐conjugated secondary antibodies and then developed using the enhanced chemiluminescence (ECL) system (Amersham Biosciences, Little Chalfont, United Kingdom). The primary antibodies used were specific for MGMT, GAPDH, IKBα, IKKα, P65, TCF1, LEF1, β‐catenin, c‐Myc, C‐Jun, HIF‐1α, EPO, and VEGF (ABCAM, Cambridge, USA).

### 2.4 Real‐time reverse transcription (RT)‐PCR

Total RNA from GBM‐XD and T98G cells was extracted using TRIzol reagent (Invitrogen Corp., Carlsbad, CA, USA) according to the manufacturer's instructions. RT was carried out with 2 μg of RNA as the template in a total volume of 20 μL with a RevertAid First Strand cDNA Synthesis Kit (Fermentas, Waltham, MA, USA). The primer sequences for the MGMT gene were as follows: forward, 5′‐GTTATGAATGTAGGAGCCCTTATG‐3′; and reverse, 5′‐TGACAACGGGAATGAAGTAATG‐3′. The amplicon size was 239 bp. Real‐time PCR was performed with the SuperScript III One‐Step RT‐PCR System (Thermo Fisher Scientific, Waltham, MA, USA), according to the manufacturer's instructions. The results are expressed as relative mRNA levels (mRNA level in FM19G1‐treated cells/mRNA level in untreated control cells).

### 2.5 Immunofluorescence staining

Tumor cells were cultured on coverslips. After the cells attached to the slips over 24 hours, FM19G11 was added to the culture media for 72 hours. The cells were then fixed with 4% formaldehyde (10 minutes), permeabilized with 0.1% Triton X‐100 for 5 minutes, and blocked with 1% BSA/10% normal goat serum for 1 hour. The cells were then incubated with anti‐MGMT antibodies (Abcam, Cambridge, USA) overnight at 4°C in the dark, followed by incubation at room temperature for 1 hour with secondary antibodies (Abcam). Then, the slips were rinsed in PBST once and in PBS twice, and mounted with DAPI mounting solution. The slips were immediately examined under a fluorescence microscope.

### 2.6 Flow cytometry

T98G cells were dissociated with 0.25% trypsin to prepare a cell suspension. The suspension was filtered through a cell strainer to remove cell clusters. The total cell number was then counted. The single‐cell suspension was centrifuged at 600 rpm for 1‐2 minutes at 4°C to remove the supernatant. The cell pellet was then resuspended in 5 mL of DMEM supplemented with 10% FBS. TMZ (500 μmol/L) and/or FM19G11 (300 nmol/L) was then added to the cells, followed by 2 hours of incubation at 37°C. The cells were then washed with cold PBS and stained with an Annexin V‐FITC/propidium iodide solution (BD Biosciences, CA, USA). The samples were analyzed on a flow cytometer (Beckman Coulter, Brea, CA, USA) using a 488‐nm excitation wavelength.

### 2.7 Bisulfite sequencing PCR (BSP)

Genomic DNA was extracted from GBM‐XD cells using a Genomic DNA Mini Tissue Kit (Invitrogen Corp.). Sodium bisulfite modification of 2 μg DNA was carried out using a MethylEasy DNA Bisulfite Modification Kit (Human Genetic Signatures, Sydney, Australia). The MGMT promoter‐associated CpG island is 267 bp in length and contains 27 CpG sites. The following primer pairs were used: F, 5′‐GGATATGTTGGGATAGTT‐3′; and R, 5′‐AAACTAAACAACACCTAAA‐3′. The PCR mixture contained 1 μL of each primer, 3 μL of bisulfite‐treated DNA, 0.8 μL of Taq DNA polymerase (4 U), and 200 mmol/L dNTPs in a final volume of 50 μL. PCR was performed with an initial denaturation step at 98°C for 4 minutes followed by 40 cycles of denaturation at 94°C for 45 seconds, annealing at 56°C for 45 seconds, and extension for 1 minute at 72°C, and a final extension at 72°C for 8 minutes. The PCR products were cleaned using a ChargeSwitch PCR Clean‐Up Kit (Invitrogen Corp.) and cloned using pUC18‐T (Sangon Biotech, Shanghai, China). The bacterial colonies containing the recombinant plasmid were amplified, and the plasmid was extracted. The plasmid was then sequenced. Ten clones were sequenced for each sample.

### 2.8 Statistical analysis

An analysis of variance was used to analyze the cell viability assay data. The chi‐square test was used to analyze the flow cytometry data. *P *<* *.05 was accepted as statistically significant.

## 3 RESULTS

**Figure 1:**
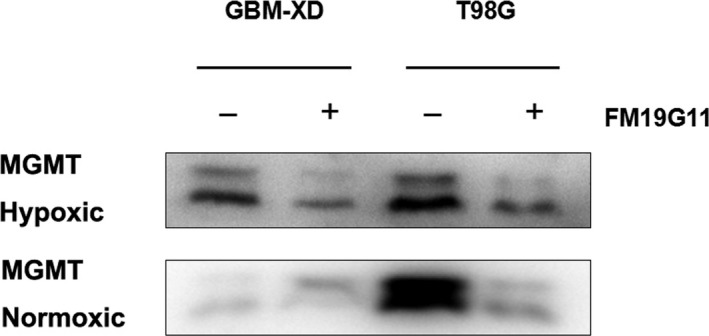
Western blotting. Both GBM‐XD and T98G cells were positive for MGMT in hypoxic culture. After FM19G11 treatment (300 nmol/L for 3 days), MGMT expression was significantly suppressed in both cell lines. In normoxic culture, T98G cells were strongly positive for MGMT, and MGMT expression was substantially downregulated by FM19G11. However, MGMT expression in GBM‐XD cells was weak, and the effect of FM19G11 on MGMT expression was unobservable

**Figure 2:**
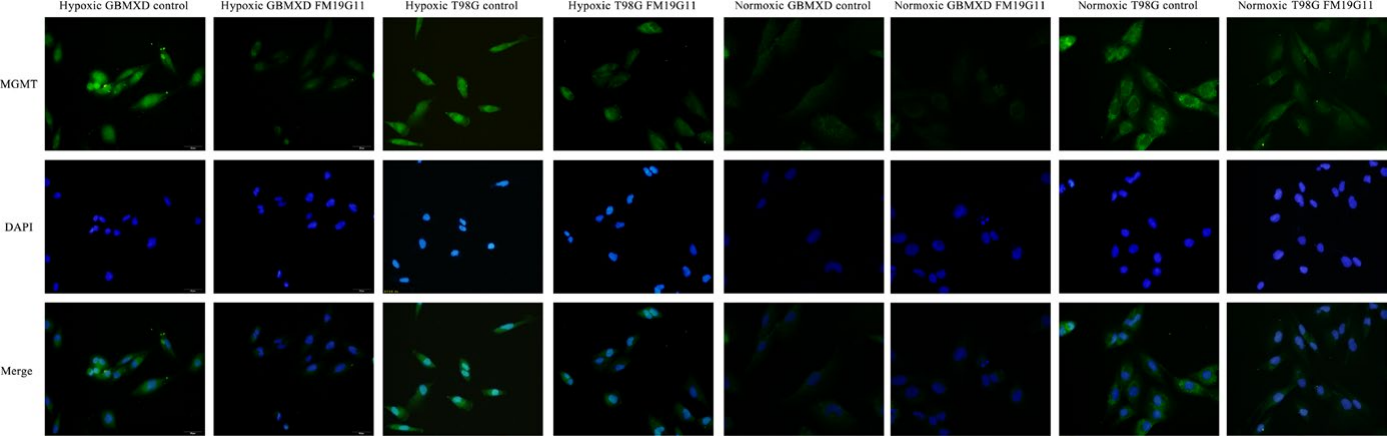
Immunocytochemistry. Both GBM‐XD and T98G cells were positive for MGMT in hypoxic culture. MGMT expression was significantly suppressed in both cell lines by FM19G11. In normoxic culture, T98G cells were strongly positive for MGMT, and MGMT expression was substantially downregulated by FM19G11. However, GMT expression in GBM‐XD cells was weak in normoxic culture

**Figure 3:**
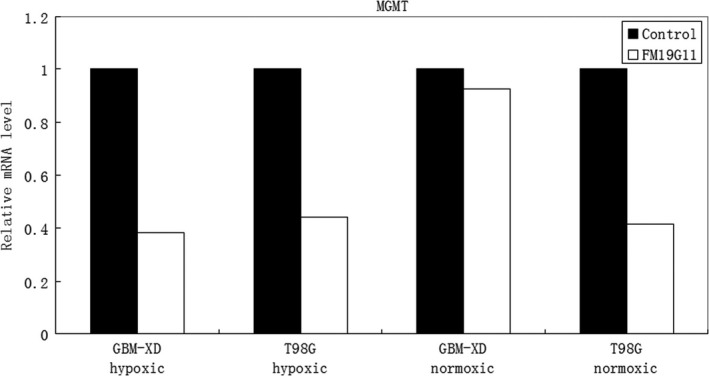
RT‐PCR. The mRNA levels of MGMT were significantly downregulated by FM19G11 treatment in hypoxic GBM‐XD, hypoxic T98G, and normoxic T98G cells

Immunocytochemistry and western blotting showed that both GBM‐XD and T98G cells were MGMT‐positive in hypoxic culture (Figures 1 and 2). After FM19G11 treatment (300 nmol/L for 3 days), MGMT expression was significantly suppressed in both cell lines (Figures 1 and 2). In normoxic culture, T98G cells were strongly positive for MGMT, and MGMT expression was substantially downregulated by FM19G11. However, MGMT expression in GBM‐XD cells was weak in normoxic culture, and the effect of FM19G11 on MGMT expression was unobservable (Figures 1 and 2). Similar to that, the mRNA levels of MGMT were significantly downregulated by FM19G11 treatment in hypoxic GBM‐XD, hypoxic T98G, and normoxic T98G cells (Figure 3).

**Figure 4:**
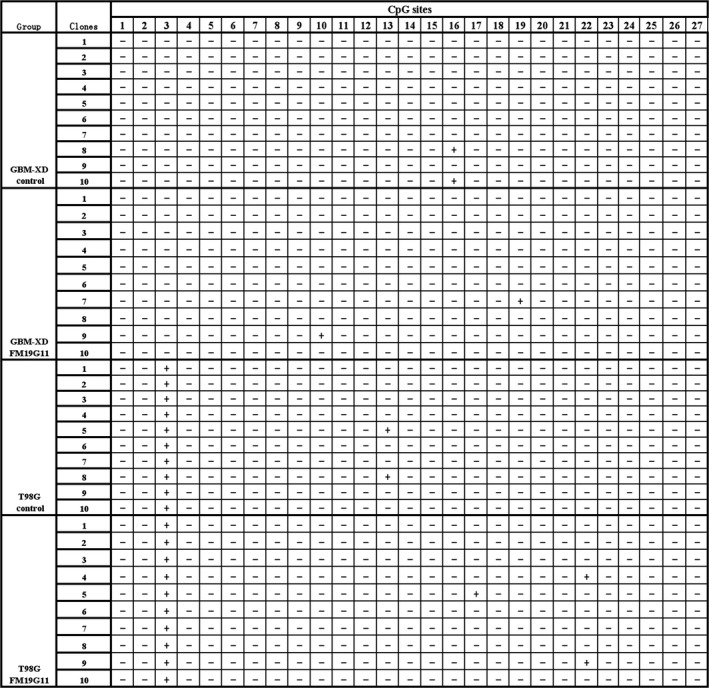
BSP. The GBM‐XD promoter was completely unmethylated, and T98G promoter had only one CpG site methylated. After FM19G11 treatment (300 nmol/L for 30 days), the promoter methylation status did not change in either cell line

As promoter methylation is the main regulator of MGMT transcription, we examined the methylation status of the MGMT promoter and found that GBM‐XD was completely unmethylated, and that T98G was partially unmethylated, with only one of the 27 CpG sites methylated. After FM19G11 treatment (300 nmol/L for 30 days), the promoter methylation status did not change in either cell line (Figure 4), suggesting that the effect of FM19G11 on MGMT expression is unrelated to promoter methylation.

**Figure 5:**
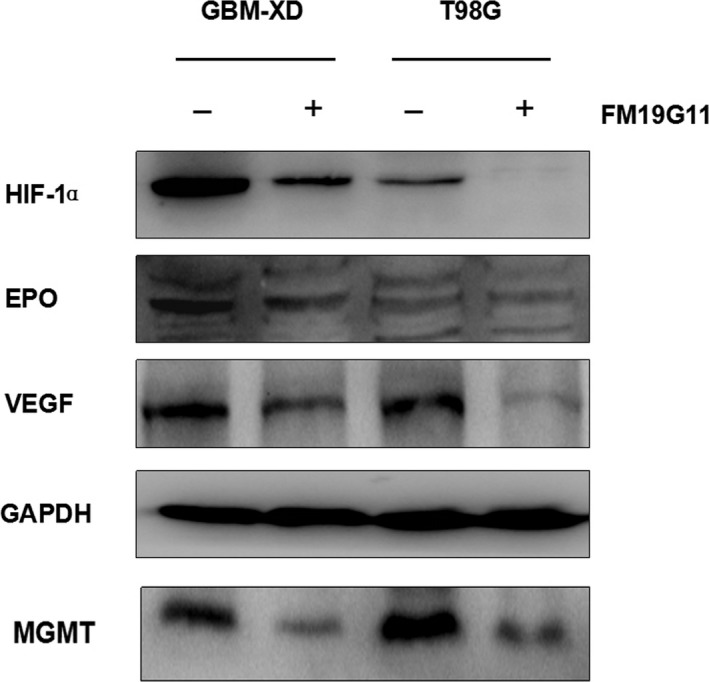
Under hypoxic conditions, HIF‐1α was expressed in both GBM‐XD and T98G cells. After FM19G11 treatment, the levels of HIF‐1α and its target genes EPO and VEGF were decreased, and the change was similar to that of MGMT

**Figure 6:**
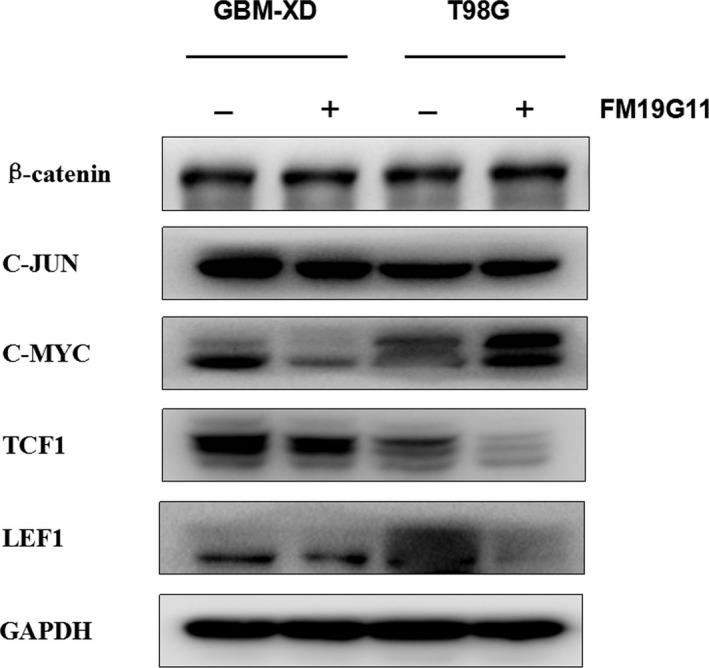
Under hypoxic conditions, the β‐catenin levels remained constant in the absence or presence of FM19G11. In addition, the related transcription factors TCF1 and LEF1 and the target genes C‐JUN and C‐MYC changed irregularly after FM19G11 treatment

**Figure 7:**
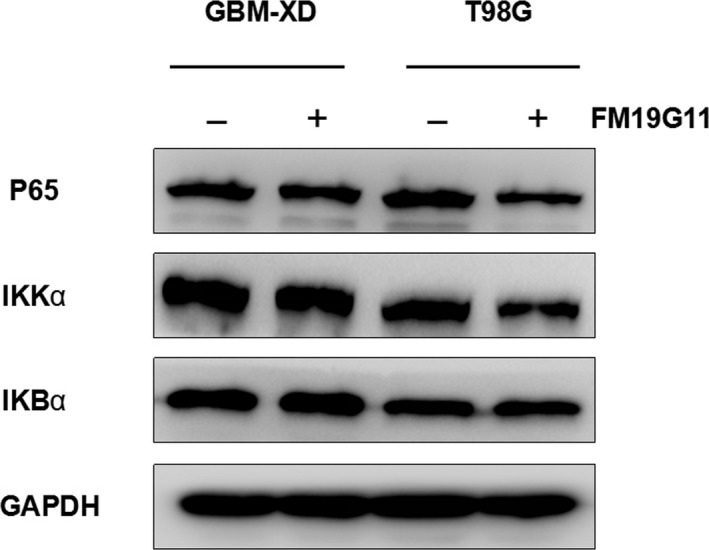
Under hypoxic conditions, P65, a member of the NF‐κB family, and the regulatory molecules IKBα and IKKα were unaffected by FM19G11

We next explored several signaling pathways that may be involved in the modulation of MGMT transcription under hypoxic conditions. HIF‐1α was expressed in GBM‐XD and T98G cells. After FM19G11 treatment, the levels of HIF‐1α and its target genes EPO and VEGF were decreased, and the change was similar to that of MGMT (Figure 5). For the WNT/β‐catenin pathway, the β‐catenin levels remained constant in the absence or presence of FM19G11. In addition, the related transcription factors TCF1 and LEF1 and the target genes C‐JUN and C‐MYC changed irregularly after FM19G11 treatment (Figure 6). For the NF‐κB pathway, we found that P65, a member of the NF‐κB family, and the regulatory molecules IKBα and IKKα were unaffected by FM19G11 (Figure 7). Taken together, we conclude that under hypoxic conditions, FM19G11 inhibits MGMT expression mainly via the HIF‐1α pathway.

**Figure 8:**
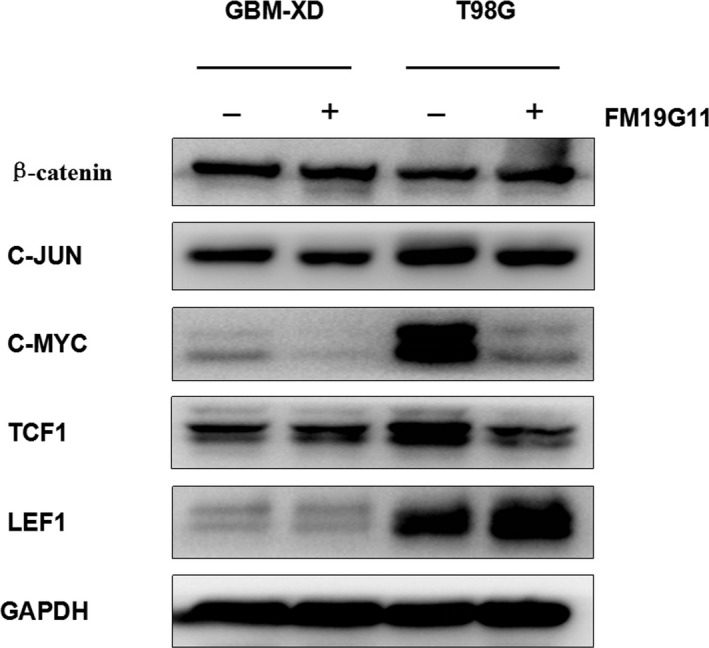
Under normoxic conditions, β‐catenin remained unchanged after FM19G11 treatment, while TCF1, LEF1, C‐JUN, and C‐MYC changed irregularly

**Figure 9:**
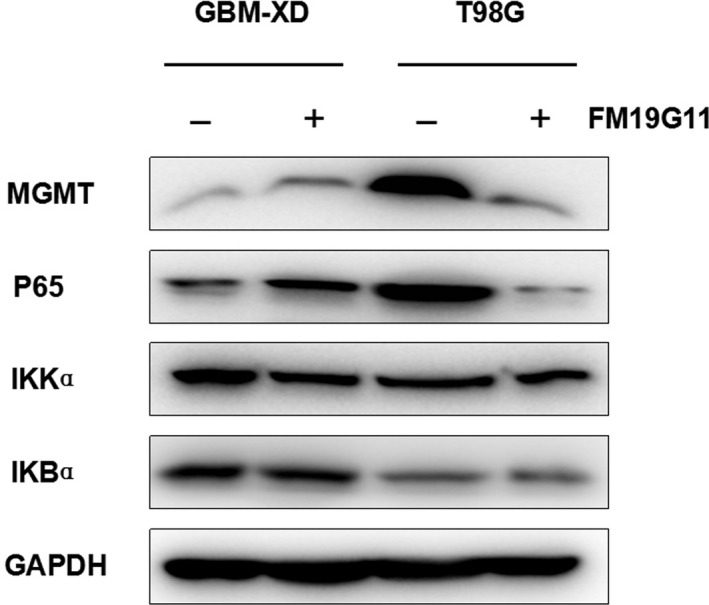
Under normoxic conditions, P65 decreased dramatically after the FM19G11 treatment of T98G cells, and the change was similar to that of MGMT. However, the regulatory molecules IKBα and IKKα did not change accordingly

Under normoxic conditions, HIF‐1α is inactivated, so we explored only the WNT/β‐catenin and NF‐κB pathways. Again, the second messenger β‐catenin remained unchanged after FM19G11 treatment, and TCF1, LEF1, C‐JUN, and C‐MYC changed irregularly (Figure 8). For the NF‐κB pathway, P65 decreased dramatically after FM19G11 treatment in T98G cells in normoxic culture, and the change was similar to that of MGMT (Figure 9). However, the regulatory molecules IKBα and IKKα did not change. These results suggest that, under normoxic conditions, FM19G11 directly inhibits NF‐κB and thus downregulates MGMT expression.

**Figure 10:**
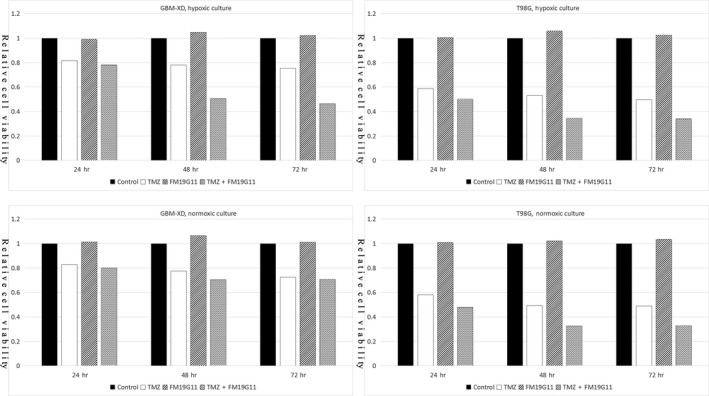
Cell viability assay. FM19G11 (300 nmol/L for 3 days) had no cytotoxicity by itself. However, FM19G11 significantly enhanced the cytotoxicity of TMZ (100 μmol/L for 3 days) in hypoxic GBM‐XD cells (TMZ group 0.754 ± 0.048 vs TMZ+FM19G11 group 0.464 ± 0.015, *P *<* *.05), hypoxic T98G cells (TMZ group 0.498 ± 0.016 vs TMZ+FM19G11 group 0.339 ± 0.009, *P *<* *.05), and normoxic T98G cells (TMZ group 0.488 ± 0.012 vs TMZ+FM19G11 group 0.327 ± 0.010, *P *<* *.05)

**Figure 11:**
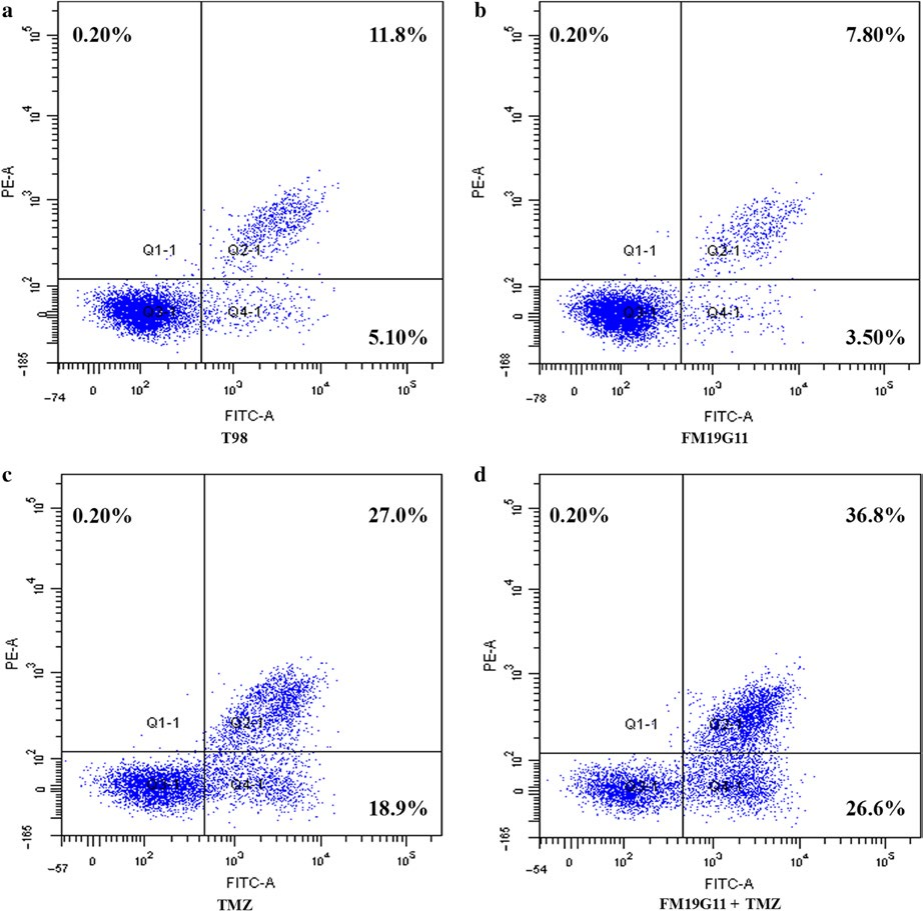
Flow cytometry. The early apoptosis percentage was 18.9% in the TMZ group and 26.6% in the TMZ+FM19G11 group (*P *<* *.05), while the late apoptosis percentage was 27.0% in the TMZ group and 36.8% in the TMZ+FM19G11 group (*P *<* *.05). In contrast, the early and late apoptosis percentages for the FM19G11 group did not exceed that of the control

As shown in Figure 10, the cell viability assay demonstrated that FM19G11 (300 nmol/L for 3 days) had no cytotoxicity by itself. However, FM19G11 significantly enhanced the cytotoxicity of TMZ (100 μmol/L for 3 days) in hypoxic GBM‐XD cells (TMZ group 0.754 ± 0.048 vs TMZ+FM19G11 group 0.464 ± 0.015, *P *<* *.05), hypoxic T98G cells (TMZ group 0.498 ± 0.016 vs TMZ+FM19G11 group 0.339 ± 0.009, *P *<* *.05), and normoxic T98G cells (TMZ group 0.488 ± 0.012 vs TMZ + FM19G11 group 0.327 ± 0.010, *P *<* *.05). Flow cytometry showed that the early apoptosis percentage was 18.9% in the TMZ group and 26.6% in the TMZ+FM19G11 group (*P *<* *.05), while the late apoptosis percentage was 27.0% in the TMZ group and 36.8% in the TMZ+FM19G11 group (*P *<* *.05). These results indicate that FM19G11 significantly enhanced the pro‐apoptotic effect of TMZ, although FM19G11 did not induce apoptosis by itself (Figure 11).

## 4 DISCUSSION

The alkylating agent TMZ is currently the standard chemotherapy for GBM.^1^, ^11^ As a prodrug, it undergoes spontaneous decomposition in solution at a physiological pH to the reactive intermediate 5‐(3‐methyl‐1‐triazeno)imidazole‐4‐carboxamide, which methylates the N^7^ and O^6^ positions of guanine and the N^3^ position of adenine.^12^ Among these 3 methylation positions, O^6^‐methylguanine (O^6^‐meG) is the most important mismatch; it results in a continuous cycle of DNA base mismatch repair with eventual strand breaks, ultimately leading to cellular apoptosis, which is how TMZ exerts its chemotherapeutic effects.^12^

However, if MGMT is expressed in tumor cells, it will directly remove the methyl group from O^6^‐meG, causing the cells to become TMZ‐resistant.^13^ Every methyl group removed from O^6^‐meG is transferred to a cysteine residue within the active site of MGMT in a stoichiometric reaction, and this MGMT molecule is then inactivated and finally degraded. This process of destruction of one MGMT molecule for each methyl group removed from methylguanine is termed suicide inhibition.^14^ Therefore, TMZ cytotoxicity is theoretically determined by the ratio of MGMT to O^6^‐meG.^14^

Many different agents have been tested for their ability to inhibit MGMT, with little success.^15^, ^16^ For example, TMZ itself was shown to partially deplete MGMT protein in tumors. In clinical practice, a dose‐dense TMZ regimen is applied to some recurrent MGMT‐unmethylated gliomas,^15^ based on the idea that a high dose of TMZ would probably deplete MGMT in tumor cells via suicide inhibition.^16^ However, these large clinical trials failed to produce an OS benefit of increasing TMZ dose intensity.^17^, ^18^ Another limitation of this strategy is that high‐dose drug exposure inevitably leads to increased side effects.^15^, ^18^

Some other chemotherapeutic agents have been tried to bypass the limitations of TMZ in MGMT‐unmethylated patients, including 1,3‐bis(2‐chloroethyl)1‐nitrosourea or 3‐[(4‐amino‐2‐methyl‐5‐pyrimidinyl) methyl]‐1‐(2‐chloroethyl)‐1‐nitrosourea hydrochloride. However, the hematological side effects of these drugs are much more frequent and severe than TMZ.^19^, ^20^

Another potential method was to sensitize glioma cells to TMZ by concomitant use of the MGMT pseudosubstrate. One potent agent is O^6^‐BG, a pseudosubstrate inactivator of MGMT. This agent has been shown to reverse resistance to TMZ by decreasing MGMT protein levels in glioma cells and animal models.^21^ However, in a clinical trial, the addition of O^6^‐BG had little therapeutic effect, and caused grade 4 hematological events in 48% of the patients, halting further attempts to use this concomitant therapy.^22^

Taken together, the above‐mentioned agents have a common drawback, namely severe side effects, which limit further study. Therefore, it is necessary to search for new agents to suppress MGMT expression, especially ones with low cytotoxicity.

FM19G11 is a small molecule agent with a molecular weight of only 463.40 g/mol.^8^ In this study, we showed that under hypoxic conditions, FM19G11 significantly inhibited MGMT expression in GBM cells by modulating the HIF‐1α pathway. However, under normoxic conditions, when the HIF‐1α pathway was inactivated, FM19G11 inhibited MGMT expression by modulating the NF‐κB pathway. As there are considerable oxygen concentration gradients in the lungs and across the capillaries and tumor tissues, the oxygen levels in the tumor should be lower than that in the air, which is defined as “normoxic condition” in this in vitro study. Therefore, under the in vivo microenvironments, HIF‐1α pathway may play a more important role than the NF‐κB pathway.

Our findings reveal that FM19G11 by itself was not cytotoxic at its effective dose (300 nmol/L for 3 days), but when FM19G11 was given concomitantly with TMZ, it strongly enhanced the pro‐apoptotic effect of TMZ. Therefore, FM1911 could be a candidate for future testing to counteract TMZ resistance in MGMT‐positive glioblastomas. There is a concern that unselective MGMT inhibition is likely to make the normal tissues, for example, the hematopoietic tissues, more vulnerable to the cytotoxicity of TMZ, and this might be a major reason for the failure of the above‐mentioned clinical trials. But as the malignant tumor tissue is generally in hypoxic state,^5^ and the MGMT inhibition effect of FM19G11 majorly relies on the hypoxia‐inducible factor‐1‐alpha pathway, we think FM19G11 probably preferentially inhibit MGMT activity in tumor than in the normal tissues. This indirect MGMT inhibition strategy may be better tolerable for the body.

## CONFLICT OF INTEREST

We have no conflict of interest. Although both TMZ and FM19G11 are from Sigma‐Aldrich, we declare that there is no conflict of interest.
